# Molecular characteristics of HBV infection among blood donors tested HBsAg reactive in a single ELISA test in southern China

**DOI:** 10.1186/s12879-020-05747-4

**Published:** 2021-01-19

**Authors:** Xianlin Ye, Tong Li, Ran Li, Heng Liu, Junpeng Zhao, Jinfeng Zeng

**Affiliations:** 1 Shenzhen Blood Center, Meigang South Road, Shenzhen, 518000 P. R. China; 2 Department of Transfusion, 2nd Affiliated Hospital, School of Medicine, Zhejiang University, Jiefang Load 88, Hangzhou, 310000 P. R. China

## Abstract

**Background:**

Hepatitis B virus (HBV) infection is a major concern for blood safety in high-prevalence HBV countries such as China. In Shenzhen, dual hepatitis B surface antigen (HBsAg) enzyme-linked immunosorbent assays (ELISAs) have been adopted in parallel with nucleic acid testing (NAT) for donors for over a decade. A small proportion of blood donors test reactive (R) for HBsAg but negative through routine NAT, which can lead to HBV infection with an extremely low viral load.

**Objectives:**

We aimed to investigate and analyze the molecular characteristics of HBV among blood donors that tested HBsAg R in a single ELISA test.

**Methods:**

Blood donations were evaluated in this study if confirmed HBsAg R through one of two ELISA kits. Samples with non-reactive (NR) results by NAT were collected and tested for HBsAg by chemiluminescent microparticle immunoassay (CLIA) with a neutralization test. The level of HBsAg was further assessed by electrochemiluminescence immunoassay (ECLIA). The viral basic core promoter (BCP) and pre-core (PC) and S regions were amplified by nested PCR. Quantitative real-time PCR (qPCR) for viral load determination and individual donation (ID)-NAT were adopted simultaneously. HBsAg was confirmed with CLIA, ECLIA, nested PCR, qPCR, and ID-NAT.

**Results:**

Of the 100,252 donations, 38 and 41 were identified as HBsAg R with Wantai and DiaSorin ELISA kits, respectively. Seventy-nine (0.077%, 79/100,252) blood samples with ELISA R-NR and NAT NR results were enrolled in the study. Of these, 17 (21.5%,17/79) were confirmed as HBsAg-positive. Of the 14 genotyped cases, 78.6% (11/14) were genotype B, and C and D were observed in two and one sample, respectively. Mutations were found in the S gene, including Y100C, Y103I, G145R, and L175S, which can affect the detection of HBsAg. A high-frequency mutation, T1719G (93.3%), was detected in the BCP/PC region, which reduced the viral replication.

**Conclusion:**

A small number of blood samples with HBsAg ELISA R-NR and NAT NR results were confirmed as HBV infection, viral nucleic acids were found in most of the samples through routine NAT methods. It is necessary to employ more sensitive and specific assays for the detection of HBV infection among blood donors.

## Introduction

According to the blood screening strategies in China, hepatitis B surface antigen (HBsAg) screening using two different enzyme-linked immunosorbent assay (ELISA) kits and a nucleic acid test (NAT) are employed for hepatitis B virus (HBV) screening to ensure blood safety for future transfusions. Blood donations are considered suitable for clinical blood transfusions when all three tests yield negative results. However, inconsistencies between HBsAg and HBV DNA results have been reported for 6–9% of blood donations [1]. Previously, we found that of 307,740 seronegative blood samples, 80 were classified as occult HBV infection (OBI), characterized by HBsAg non-reactive (NR) and NAT reactive (R) [2].

Another consideration is HBsAg R and NAT NR results. More specifically, blood with HBsAg ELISA R-NR and NAT NR results may place recipients at risk of HBV transmission, due to the extremely low levels of HBsAg and HBV DNA, respectively. Previous studies have shown that mutations in the S region and basic core promoter (BCP) and pre-core (PC) of HBV can affect the detection of HBsAg and viral nucleic acid [3, 4].

To accurately determine the presence of HBV infection in blood donors, we conducted blood screening for HBV DNA and HBsAg using ELISA, chemiluminescent microparticle immunoassay (CLIA), and electrochemiluminescence immunoassay (ECLIA). Furthermore, quantitative real-time PCR (qPCR) and nested PCR were performed to investigate the molecular characteristics of HBV with low viral loads among blood donors that tested HBsAg R in a single ELISA test, and to identify the potential molecular mechanisms underlying this mode of HBV infection.

## Materials and methods

### Study samples

Between February 2017 and February 2018, a total of 100,252 blood donations from Shenzhen Blood Center underwent HBsAg testing using two different ELISA kits (Beijing Wantai Biological Pharmacy, Beijing, China; DiaSorin, Saluggia, Italy), and HBV DNA using the Procleix Ultrio Plus Assay (Grifols, Novartis Vaccines and Diagnostics, Inc. Spain; 0.5 mL, LOD: 3.4 IU/mL), of which 363 (0.36%, 363/100,252) donors with HBV reactive results were found, including 221 HBsAg ELISA R-R, 63 ELISA NR-NR and NAT R, and 79 HBsAg ELISA R-NR/NAT NR. All 79 samples with ELISA R-NR and NAT NR results were investigated in this study.

### HBsAg confirmation

All samples with HBsAg ELISA R-NR and NAT NR results underwent HBsAg confirmatory testing; samples evaluated in the study were subjected to HBsAg screening using CLIA (ARCHITECT HBsAg Reagent Kit, Abbott Ireland Diagnostics Division Finisklin Business Park, Sligo, Ireland) and ECLIA (Elecsys HBsAg II, Roche Diagnostic GmbH, Mannheim, Germany). Then, samples with CLIA HBsAg reactive results were subjected to a neutralization test using a kit provided by the same manufacturer (Abbott). Following CLIA and ECLIA testing, samples were further tested for HBV DNA using standard methods (qPCR and nested PCR) [5, 6]. In addition, individual donation NAT (ID-NAT) (Cobas TaqScreen MPX Test, version 2.0, Roche Diagnostic GmbH, Mannheim, Germany; 1.0 mL, LOD, 2.3 IU/mL) was used for the detection of HBV DNA. HBsAg-positive results were confirmed by reactivity in CLIA and/or ECLIA, as well as any reactive results in qPCR, nested PCR, and ID-NAT [7].

**Fig. 1:**
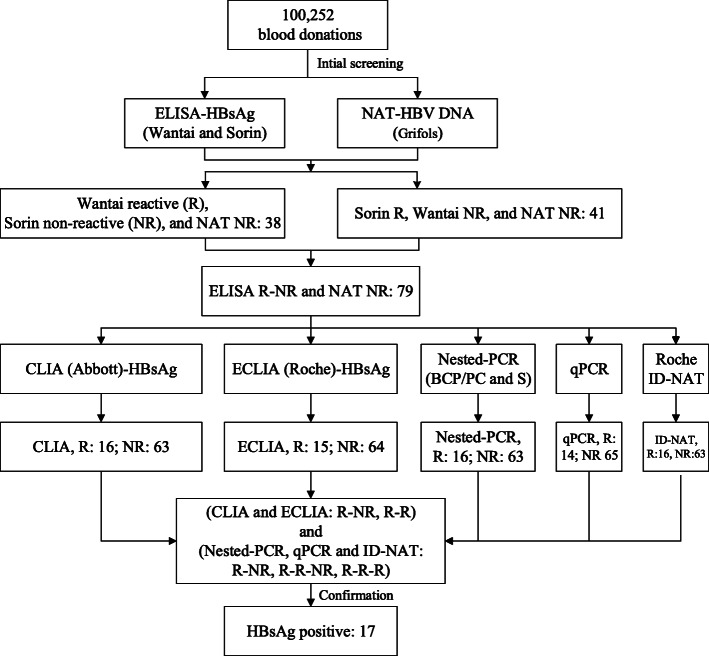
The study route, R: reactive, NR: non-reactive, Wantai: Diagnostic Kit for Hepatitis B Virus Surface Antigen (ELISA), Sorin: Murex HBsAg version3, NAT-Roche: Cobas TaqScreen MPX Test, version 2.0, NAT-Grifols: Procleix Ultrio Plus Assay, CLIA: ARCHITECT HBsAg Reagent Kit, ECLIA: Elecsys HBsAg II

Viral nucleic acid was extracted from 2.5 mL plasma using the HighPure Viral Nucleic Acid Large Volume Kit (Roche Diagnostics GmbH, Mannheim, Germany), and nested PCR was performed to amplify the BCP/PC regions (263 bp) and S-fragment (496 bp) as previously described [6]. The nested PCR products were purified and subjected to Sanger sequencing by Shenzhen BGI Technology Co., Ltd. Following nucleic acid extraction, the viral load of HBV was determined using qPCR [5]. The confirmation algorithm is shown in Fig. 1.

### HBV phylogenetic analysis and mutations in the BCP/PC and S regions

**Fig. 2:**
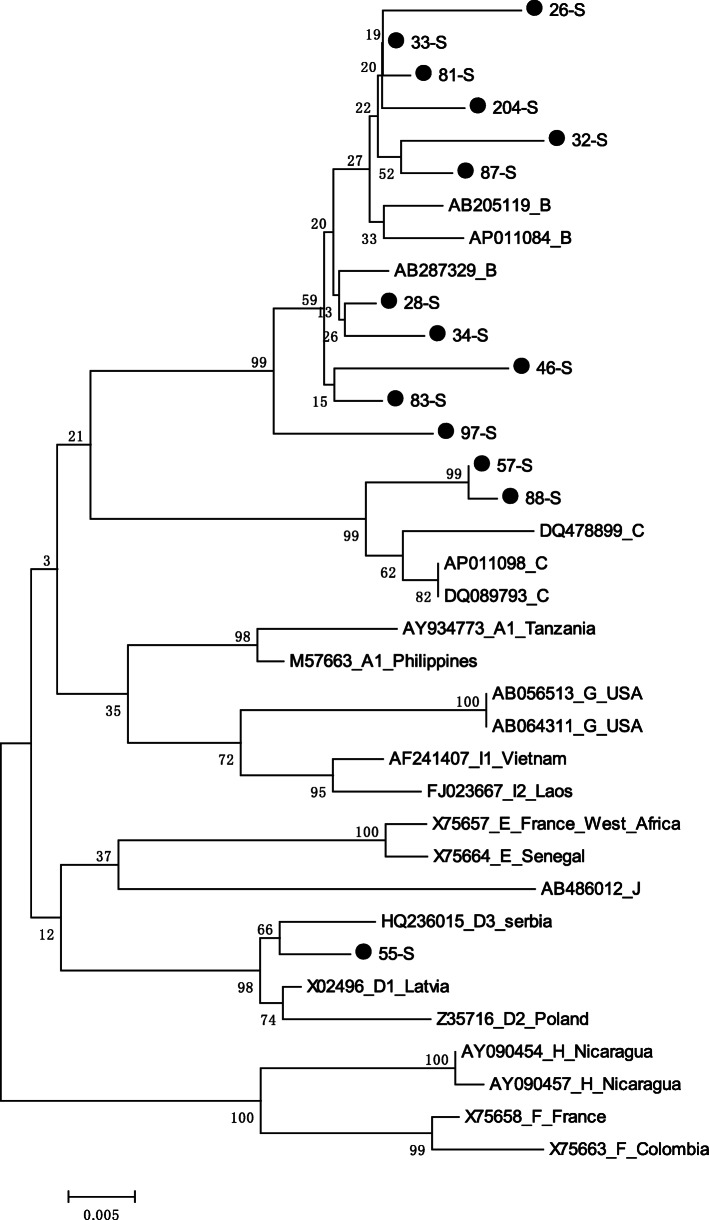
Phylogenetic tree analysis of S region sequences among 14 HBV isolates in blood donors

The sequences were aligned and compared with HBV reference sequences of genotype A-I (accession numbers are shown in Fig. 2) using the BioEdit program. Nucleotide alignments (S region) were used to build a phylogenetic tree for HBV subtyping by MEGA software using the neighbor-joining algorithm based on the Kimura 2-parameter model for 1000 bootstrap replicates. The mutations in the BCP/PC and S regions were analyzed and described in a previous study [6]. We matched the amino acid sequences of blood donors with the reference sequences and calculated the frequency of amino acid mutations.

### Statistical analysis

Demographic data were obtained from the donor/donation database from the blood center. SPSS 21.0 software was used for statistical analysis.

## Results

### HBsAg confirmation and demographic characteristics of blood donors

Among the 79 blood samples with HBsAg ELISA R-NR and NAT NR results in initial tests, 17 (21.5%, 17/79) were confirmed as HBsAg-positive (Fig. 1). Wantai (ELISA) reported 38 reactive results (S/CO range: 1–21.89, median: 1.58) and DiaSorin (ELISA) reported 41 reactive results (S/CO range: 1–6.87, median: 1.23). Of the HBsAg-positive samples, the median HBsAg titer was 1.55 IU/mL, and only two samples were below the LOD (0.05 IU/mL) of HBsAg in the ECLIA. All 17 HBsAg-positive samples were NAT R. Detectable viral load was reported for 14 of the 79 (17.7%) samples, including 5 of 10–100 IU/mL, 7 of 100–1000 IU/mL, and 2 over 1000 IU/mL viral load, after the qPCR test. The viral load ranged between 2649.5 IU/mL and 12 IU/mL (median, 128.5 IU/mL; Table 1). Of the 17 HBsAg-positive samples with NR results for initial NAT screening (Grifols), 14 (82.4%) and 16 (94.1%) were detected by qPCR and MPX ID-NAT (Roche), respectively.

**Table 1:** Initial screening and confirmatory tests of HBsAg and demographic characteristics

ID	Initial Screening (LOD)			Confirmatory test (LOD)										Demographic information		
	NAT Grifols (3.4 IU/ml)	ELISA-HBsAg		CLIA-HBsAg (0.05 IU/mL)			ECLIA-HBsAg (0.05 IU/mL)		Nested-PCR (10 IU/ml)		qPCR IU/mL (5 IU/mL)	Roche ID-NAT (2.3 IU/ml)	Final result	Age	Sex	Donation
		Wantai 0.1 IU/ml)	Sorin (0.05 IU/ml)	S/CO	Result	Neutralization test result	Titer	Result	BCP	S-subtype						
26	–	0.18	1.3	14.01	+	+	0.50	+	+	B	128.5	+	Positive	49	Male	Repeat donor
33	–	0.06	1.03	5675.14	+	+	398.70	+	+	B	514	+	Positive	40	Female	Repeat donor
34	–	0.21	1.1	30.47	+	+	1.55	+	+	B	536	+	Positive	48	Female	First-time donor
55	–	0.19	2.13	5.08	+	+	0.08	+	+	D	12	+	Positive	27	Male	First-time donor
80	–	0.04	6.87	58.71	+	+	1.64	+	–	–	116.4	+	Positive	49	Male	Repeat donor
81	–	0.52	1.52	530.64	+	+	11.65	+	+	B	83.2	+	Positive	27	Female	First-time donor
87	–	0.33	1.13	3.09	+	+	0.06	+	+	B	49.6	+	Positive	26	Male	First-time donor
88	–	0.05	1.23	422.21	+	+	14.98	+	+	C	–	+	Positive	41	Female	Repeat donor
95	–	0.06	1.39	12.64	+	+	0.53	+	+	–	407	+	Positive	32	Female	First-time donor
97	–	0.68	1.93	765.92	+	+	18.71	+	+	B	2654.3	+	Positive	33	Male	First-time donor
219	–	0.51	1.61	1.31	+	+	< 0.05	–	+	–	–	–	Positive	33	Male	First-time donor
28	–	2.11	0.51	45.27	+	+	2.06	+	+	B	378.6	+	Positive	27	Female	Repeat donor
32	–	1.03	0.5	4.78	+	+	0.46	+	+	B	74.5	+	Positive	23	Male	First-time donor
46	–	1.06	0.50	3.23	+	+	0.18	+	+	B	14.1	+	Positive	31	Female	Repeat donor
57	–	1.08	0.51	0.66	–	–	0.05	+	+	C	34.8	+	Positive	22	Male	First-time donor
83	–	1.41	0.42	246.90	+	+	10.97	+	+	B	1373	+	Positive	24	Male	First-time donor
204	–	4.13	0.45	1.23	+	+	< 0.05	–	–	B	–	+	Positive	37	Female	Repeat donor

Most HBsAg-positive patients (64.7%, 11/17) were 18–35 years old and first-time blood donors (58.8%, 10/17). Nearly half of the blood donors were female (47.1%; Table 1).

### HBV genotype classification

The S fragments of 14 samples were successfully sequenced. A phylogenetic tree of the S sequence of HBV was constructed using MEGA software based on the Kimura-2 parameter model with 1000 bootstrap replicates (Fig. 2). Eleven cases (78.5%, 11/14) of HBV-B, two cases (14.3%, 2/14) of HBV-C, and one case (7.1%, 1/14) of HBV-D were identified among the 14 isolates.

### Mutations in S and BCP/CP sequences

A total of 32 mutations in the S sequence were observed in 13 blood donors. As shown in Table 2, 45.5% (5/11, 45.5%) S regions in 11 HBV-B isolates had N40S mutations. Several mutations associated with the interference of HBsAg detection, such as mutations in the major hydrophilic region (MHR), including Q129H, T131I, M133L/S/T, F134L, T143M, and G145R, and mutations outside the MHR, including Y100C, L175S, and Y103I, were found in S genes among HBV-infected blood donors. In addition, immune escape mutants containing Q129H, T131I/T, G145R, and E164V were also found in these S sequences.

**Table 2:** Mutation analysis in S and BCP/PC sequences

Donor ID	Genotype	Mutation site		qPCR IU/mL	HBsAg test results			
		S region	BCP/PC region		Wantai ELISA	Sorin ELISA	Abbott CLIA	Roche ECLIA
26	B	Y100C, F134L, G145R	T1719G, C1858T, G1896A	128.5	–	+	+	+
28	B	N40S	T1719G, A1846T, C1858T, C1913A	378.6	+	–	+	+
32	B	I86T, Q129H, T131I, M133S, Y161H	T1719G, T1754G, C1858T	74.5	+	–	+	+
33	B	None	T1719G, A1752G, T1800C, T1815C, C1877T, C1858T	514	–	+	+	+
34	B	N40S, P46T	T1719G, C1858T, A1874G	536	–	+	+	+
46	B	N40S, K122R, Y161F	T1719G, A1752G, C1858T	14.1	+	–	+	+
55	D	M131I, I150M	T1727A, G1757A, T1802C, C1858T, G1915T	12	–	+	+	+
57	C	G96V	T1719G, G1721A, T1727A, G1757A, A1775G, C1858T	34.8	+	–	–	+
80	–	–	–	116.4	–	+	+	+
81	B	S55F	T1719G, A1762T, G1764A, A1775G, C1858T	83.2	–	+	+	+
83	B	N40S	T1719G, A1846T, C1858T, A1874G	1373	+	–	+	+
87	B	M103I, M133T	T1719G, C1858T	49.6	–	+	+	+
88	C	G96V	None	–	–	+	+	+
95	–	–	T1719G, A1762T, G1764A, A1808G, C1817T, C1858T	407	–	+	+	+
97	B	N40S, L49P, S55F, S58C, C64S, C76F, T143M	T1719G, T1754G, C1858T	2654.3	–	+	+	+
204	B	S174N, K122R, E164V	–	–	+	–	+	–
219	–	–	T1719G, C1858T	–	–	+	+	–

–: Non-reactive, +: Reactive, None: no mutation

Twenty-one mutations were identified in the core upstream regulatory sequence (nt1678–1741) from 16 samples, and T1719G occurred in 93.8% (15/16) samples. The most common mutations in the BCP region (nt1742–1849) and pre-C region were A1752G and A1846T. The A1762T and G1764A mutations were found in two cases. In addition, two insertion mutations were observed in nt1847 and nt1915 (insertion of base C).

## Discussion

HBV infection remains a major threat to public health and blood safety. In China, approximately 50% of the individuals have a history of HBV infection, and 7.2% are chronic carriers of HBsAg [8]. HBsAg and HBV DNA screening were implemented among blood donors to decrease the risk of HBV transmission through blood transfusion [9]. Complicated infectious modes, such as OBI, HBsAg ELISA R-NAT NR, and ELISA R for HBV-associated markers, have been reported in Shenzhen blood donors [7, 10]. The occurrence of such complications restricts the reliability of HBV detection in China. Notably, samples with HBsAg ELISA R-NR and NAT NR results may have a risk of HBV transmission, due to the low levels of HBsAg and HBV DNA. Due to the blood screening protocol in China, limited data on the molecular diversity and mutation analysis of HBV in blood donors is currently available. Hence, we investigated the molecular characteristics of HBV in blood donors that were missed by initial NAT screening but detected as HBsAg ELISA R-NR through two different ELISA kits.

In this study, 79 samples with HBsAg ELISA R-NR and NAT NR results were found in 100,252 blood donations. After a series of confirmatory tests, of which 17 of 79 (21.9%) samples were confirmed as HBsAg-positive, accounting for 0.017% (17 of 100,252) of the overall sample pool (95%CI: 0.0097–0.026%). A previous study reported that 10% of blood samples with HBsAg ELISA R-NR results were transferred to ELISA R-R results after follow-up studies [11], and HBV DNA was detected in 13.3% (4/30) of such ELISA R-NR samples using qPCR [12], which was lower than that in our results (21.9%). This discrepancy may be due to manufacturer differences.

Seventeen samples were missed by one of the two ELISA kits, but most of them were detected by CLIA (16) and ECLIA (15). Currently, ELISA is the only serological assay approved for HBV blood screening in China. Automated CLIA and ECLIA have been used in clinical laboratories but not for HBV screening among blood donors. Furthermore, of the 17 HBsAg-positive samples with NR results for initial NAT screening (Grifols), most HBV infections were detected by nested PCR (16) and qPCR (14). Previously, we identified 14 OBI patients (HBsAg ELISA NR and NAT R) out of 490 donors, who tested ELISA reactive for hepatitis B core antibodies (HBcAb), after the application of more sensitive assays [13]. Hence, more sensitive serological and NAT assays are needed for blood screening.

In addition to the different capabilities available for HBV detection, HBV genetic diversity and various mutations should be considered when investigating inconsistencies in HBV screening assays. The majority of HBV-positive blood donors in this study were genotype B (11/13, 84.6%), which is similar to our previous report in Shenzhen in 2015 (14/15, 93.3%) [8]. A nationwide survey reported that HBV-B was predominant in the southern areas of China [14]. Genotype D is mainly found in the Mediterranean region and in certain Asian countries [15]. One genotype D strain observed in our study highlights that increased international travel and immigration may have contributed to the input of the epidemic.

HBV is prone to mutations in various gene regions. Typically, mutations in the HBsAg region can cause immune escape, resulting in the failure to detect HBsAg [16]. Furthermore, HBV immune escape mutants are highly contagious and pathogenic in immunodeficient patients [17]. In vivo studies have found that certain mutations in the S region of HBV can lead to reduced, or even inhibited, secretion of viral particles, and reduced HBsAg synthesis [18]. Mutations in the MHR and non-MHR in the S region, such as Y100C, Y103I, Q129H, T131I, M133L/S/T, F134L, T143M, G145R, and L175S, were found in this study, and could reduce the affinity of monoclonal antibodies for “a” epitopes by altering the structure of HBsAg protein [19], which may contribute to the failure of commercial reagents.

Mutations in the BCP/PC region can regulate the secretion of the hepatitis e antigen (HBeAg), leading to the cessation of antigen production. Typically, BCP mutations (A1762T and G1764A) can reduce the mRNA synthesis of the pre-C region, which is reflected as a very low level of HBV DNA [20]. G1896A is another typical mutation in the pre-C region, which generates a stop codon at the 28th amino acid position of the HBeAg sequence, resulting in the inhibition of protein synthesis [21]. These three mutations in the BCP/PC region found in this study are closely associated with the development of hepatocellular carcinoma [20]. Fifteen cases of T1719G mutations in the BCP/PC region were identified in 16 samples in the present study, which can inhibit in vitro HBV replication [22], and may lead to a low HBV DNA viral load.

It is worth noting that among the 17 samples with HBV infection, two samples (ID: 83, 97) had high viral loads (> 1000 IU/mL) detected by qPCR, but were reported as NR in routine NAT testing (Grifols). The N40S mutation in the S region was identified in both samples. Whether this has an impact on NAT requires further investigation. Some studies have reported false-negative results and underestimated viral load among blood donors with HBV infection using various commercial kits [23, 24]. Therefore, NAT with two targets in different gene regions is recommended to avoid mismatch due to possible mutations.

In summary, we analyzed the molecular characteristics of HBV among blood donors using routine ELISA R-NR and NAT NR results. Most positive cases were identified by CLIA and ECLIA, as well as qPCR and nested PCR. Analysis of the S gene sequence revealed a large number of mutations, which are associated with disrupting HBsAg detection. Some mutations in the BCP/PC regions were identified in this study, which may lower the viral load of HBV DNA and cause false-negative NAT results. Our results highlight the need to improve the sensitivity of HBsAg screening and NAT methods, to aid in the detection of low HBV viral loads and mutated HBV strains.

## Data Availability

The data for this study is available from the corresponding author on reasonable request.
